# Designing the selenium and bladder cancer trial (SELEBLAT), a phase lll randomized chemoprevention study with selenium on recurrence of bladder cancer in Belgium

**DOI:** 10.1186/1471-2490-12-8

**Published:** 2012-03-21

**Authors:** Maria E Goossens, Frank Buntinx, Steven Joniau, Koen Ackaert, Filip Ameye, Ignace Billiet, Johan Braeckman, Alex Breugelmans, Jochen Darras, Kurt Dilen, Lieven Goeman, Eliane Kellen, Bertrand Tombal, Siska Van Bruwaene, Ben Van Cleyenbreuge, Frank Van der Aa, Kris Vekemans, Hendrik Van Poppel, Maurice P Zeegers

**Affiliations:** 1 Department of General Practice, University of Leuven, ACHG-KU Leuven, Kapucijnenvoer 33, Blok J, bus 7001, 3000 Leuven, Belgium; 2 Unit of Urologic and Genetic Epidemiology, University of Birmingham, Birmingham, UK; 3 Universiteit Maastricht, Maastricht, The Netherlands; 4 Department of Urology, UZ Leuven, Leuven, Belgium; 5 Department of Urology, Sint-Elisabethziekenhuis, Turnhout, Belgium; 6 Department of Urology, AZ Maria Middelares, Ghent, Belgium; 7 Department of Urology, AZ Groeninge, Kortrijk, Kortrijk, Belgium; 8 Department of Urology, UZ Brussel, Brussel, Belgium; 9 Department of Urology, Heilig Hart ziekenhuis, Leuven, Belgium; 10 Department of Urology, AZ Damiaan, Oostende, Belgium; 11 Department of Urology, Jessa ziekenhuis, Hasselt, Belgium; 12 Department of Urology, Kliniek Sint-Jan, Brussel, Belgium; 13 Leuven University Centre for Cancer Prevention (LUCK), Department of Urology, Cliniques universitaires Saint-Luc, Brussel, Belgium; 14 ACHG-KULeuven, Kapucijnenvoer 33 - Blok J - bus 7001, 3000 Leuven, Belgium

**Keywords:** Selenium, Bladder cancer, Transitional Cell Carcinoma, Chemoprevention, Randomized clinical trial, Urology

## Abstract

**Background:**

In Belgium, bladder cancer is the fifth most common cancer in males (5.2%) and the sixth most frequent cause of death from cancer in males (3.8%). Previous epidemiological studies have consistently reported that selenium concentrations were inversely associated with the risk of bladder cancer. This suggests that selenium may also be suitable for chemoprevention of recurrence.

**Method:**

The SELEBLAT study opened in September 2009 and is still recruiting all patients with non-invasive transitional cell carcinoma of the bladder on TURB operation in 15 Belgian hospitals. Recruitment progress can be monitored live at http://www.seleblat.org. Patients are randomly assigned to selenium yeast (200 μg/day) supplementation for 3 years or matching placebo, in addition to standard care. The objective is to determine the effect of selenium on the recurrence of bladder cancer. Randomization is stratified by treatment centre. A computerized algorithm randomly assigns the patients to a treatment arm. All study personnel and participants are blinded to treatment assignment for the duration of the study.

**Design:**

The SELEnium and BLAdder cancer Trial (SELEBLAT) is a phase III randomized, placebo-controlled, academic, double-blind superior trial.

**Discussion:**

This is the first report on a selenium randomized trial in bladder cancer patients.

**Trial registration:**

ClinicalTrials.gov identifier: NCT00729287

## Introduction

In Belgium, 2159 patients were newly diagnosed with a primary bladder cancer in 2008. These numbers have been stable over the last five years. The incidence and mortality rates increase sharply with age and about two-thirds of patients are ≥ 65 years old. The mean age of diagnosis is 73 years for men and 74 years for women. In the age group 45-59 years, the male/female ratio is 3:3, while in the age group 60-74 years the incidence rates in males are more than fivefold than the rates in females. The relative 5-year survival is 47% for men and 54% for women [1]. Almost 400,000 bladder cancer cases occurred worldwide in 2008 [2]. Moreover, in the USA and probably in most Western countries, bladder cancer is the most expensive cancer in terms of healthcare expenditure [3] because of lifetime ongoing cystoscopies. Any reduction in the need for cystoscopies reduces the cost and even more important, improves quality of life.

Evidence supporting the use of selenium as a general cancer preventive agent includes proof from geographical, animal, in vitro, epidemiological and intervention studies. High selenium intake in Venezuela is associated with a reduced cancer risk [4]. In animal models, antitumourigenic activity has been observed for metabolites of naturally occurring forms of selenium such as selenomethionine, selenocysteine and methylselenocysteine and inorganic selenium salts, such as selenite and selenate [5,6]. Recent in vitro studies have demonstrated that selenium may be an effective chemopreventive and anticancer agent with a broad spectrum against several human cancer cells (prostate, colon, bladder, lung, liver, ovarian, leukemia). In total, twenty-eight different selenium compounds have been reported to have anticancer, chemopreventive or apoptotic activities [7-9]. Three case-control studies reported an increased risk of bladder cancer, associated with lower serum [10] and toenail [10-12] selenium concentrations. A meta-analysis of bladder cancer incidence in five observational studies [13-17] found an inverse association with an overall risk estimate of 0.67 (95% CI 0.46 to 0.97) suggesting a protective effect of higher selenium levels against bladder cancer [18]. The Nutritional Prevention of Cancer (NPC) study from Clark in 1996 was the first intervention study that showed a decrease in the incidence of prostate, lung and colorectal cancers in the selenium-supplemented group of older Americans. The effect seemed to be the strongest in the individuals with the lowest selenium status (< 123.2 ng/mL) [19,20]. Nevertheless, not all cancer prevention trials indicated reduced cancer risk by selenium supplementation. The selenium and Vitamin E Cancer Prevention Trial (SELECT) failed to show a benefit of selenium supplementation in reducing the risk of prostate cancer in a population of healthy men [21]. The lack of positive effect of selenium supplementation on the prostate cancer incidence observed in this study may have been due to the different form of selenium used, selenomethionine, compared to Se-enriched yeast in the NPC study [8,22].

The review of Brinkman et al. [23] and the later studies of Grossman et al. [24], Altwein et al. [25], Busby et al. [26] and Amaral et al. [27] suggest that selenium may be suitable for chemoprevention as well as for treatment. It is useful to perform a selenium trial in a country such as Belgium where the selenium intake is low due to low soil selenium content. In the Belgian case control study the mean blood selenium level in the cases was 78.77 ng/mL compared with 92.31 ng/mL in the controls [10]. This contrasts with the patients enrolled in the NPC and the SELECT trials who had higher initial plasma levels of selenium (113 ng/mL and 135 ng/mL, respectively) [28] and an intake of approximately 90 μg/day. As selenium is mainly excreted in the urine, it comes into direct contact and has prolonged exposure with the bladder mucosa, making the role as a potential chemoprevention agent biologically plausible, unlike the case in prostate cancer.

The objective of SELEBLAT is to investigate whether 200 μg/day Selenium-yeast, in addition to standard care, reduces the risk of recurrence for patients with non-invasive bladder cancer.

## Methods

### Clinical design

SELEBLAT is a phase III randomized, double-blind, placebo-controlled, multicentre, academic trial with 200 μg/day Selenium-yeast supplementation during 3 years with a subsequent follow-up period of 3 years aimed at the prevention of recurrence of non-muscle-invasive bladder cancer (NMIBC). SELEBLAT was initiated by the Department of General Practice of the University of Leuven and the Department of Urology of the University hospitals of Leuven, Belgium with funding by the Agency for Innovation by Science and Technology Belgium (IWT).

#### Outcome

The primary endpoint of SELEBLAT is the recurrence-free interval defined as the time from the date of trial entry to the date of recurrence in patients with superficial transitional cell carcinoma of the bladder. A recurrence is defined as the new occurrence of a bladder cancer at the same or at a different site as the index cancer and excluding tumours identified at the first cystoscopy at three month. Tumours identified within the first three months are considered as incompletely resected primary tumours in patients who were macroscopically tumour-free after the first resection.

A secondary endpoint is the progression-free interval defined as the time from the date of trial entry to the date of progression. Progression is defined as a recurrence with an increase in tumour grade from low grade (G1-G2) to high grade (G3), or an increase in TNM stage, or a new occurrence of carcinoma in situ (CIS) in the bladder previously free from such lesions, or a new occurrence of multiple tumours following resection of a solitary tumour, or the need for a cystectomy because of refractory disease.

#### Eligibility criteria for participants

Men and women, at least 18 years of age, who give informed consent, are eligible for inclusion in SELEBLAT, if they have undergone a transurethral resection (TUR) of a histologically confirmed transitional cell carcinoma of the bladder, stage pTa, pT1 or Carcinoma in situ (Cis). Primary and second primary tumours are included. Eligibility and exclusion criteria are:

• Men and women, aged above 18 years

• Histologically confirmed non-muscle-invasive transitional cell carcinoma (< pT2): stage pTa, pT1 or Carcinoma in situ (Cis)

• Primary and second primary tumours

• Able to be randomized within three months after transurethral resection of the bladder tumour

• Must be able to swallow pills

• Must agree not to take supplements containing selenium, apart from the trial medication

• Fertile female patients must use effective contraception

• No invasive or metastatic disease (stage pT2 or above)

• No history of any type of malignancy within the past five years

• No other serious medical or psychiatric illness that would preclude giving informed consent

• No known hypersensitivity or adverse reactions to selenium

• Not taking more than 50 mg selenium/day as a dietary supplement, including multivitamin supplements within the last 30 days

• Not concurrently participating or having participated in any other clinical trial involving a medical, surgical, nutritional or life-style intervention (unless no longer receiving the intervention and being in the follow-up phase)

#### Participant recruitment

Patients are recruited by the participating urologists of 15 hospitals throughout Belgium (Flanders and Brussels) from September 2009 to August 2012. All subjects have to master the Dutch or French language and may be excluded due to illness, mental sickness or incapacity to comprehend the questions as evaluated by the treating clinician. Eligible patients receive oral and written information (information brochure) from the research nurse and sign an informed consent form. The procedures followed are in accordance with the ethical standards of the responsible committee on human experimentation (institutional and national) and with the Helsinki Declaration of 1975, as revised in 2008. The study has been approved by the ethical review board of the University Hospital of Leuven acting as the central ethical review board, by the trial steering committee and by the appropriate ethical review boards related to the hospitals in which it is performed. A Data Safety Monitoring Board (DSMB), comprised of individuals with expertise in the areas of medicine and biostatistics, has been established to serve as an external review committee to monitor the progress of the study including accrual and adverse events.

#### Randomization

Randomization is stratified by treatment centre. Once a patient is deemed to be eligible for the trial and has given written consent, a trained research nurse randomizes the patient by using the database. A computerized algorithm randomly assigns the patients to a treatment arm. All study personnel and participants are blinded to treatment assignment for the duration of the study. To evaluate patient blinding, patients are asked by a questionnaire to evaluate which treatment they believe they have received (selenium, placebo, or don't know). If patients answer either selenium or placebo, they are asked to indicate what led to that belief.

#### Participant follow-up

Study participants are supplied with the study drug and followed on a bi-annual basis for a period of up to three years. This follow-up phase entails questionnaire distribution and monitoring for adverse events. Study questionnaires capture the onset of new illnesses and symptoms, including potential selenium-related toxicities.

#### Investigational medicinal product (IMP)

The active product is selenium (200 μg/day, in the form of high-selenium yeast, SelenoPrecise from PharmaNord, Vojens, Denmark). A six-month intake of 200 μg of SelenoPrecise yeast increases the plasma selenium levels on average from a baseline value of (mean ± SD) 92 ± 20 ng/mL to 196 ± 42 ng/mL. The increase of the plasma selenium level is dose-dependent [29]. Patients in each study arm received an oral tablet (selenium or placebo) to be taken daily, in addition to standard care. Standard care is provided as described by Oosterlinck in the "Guidelines on diagnosis and treatment of superficial bladder cancer" [30]. Placebos have been manufactured to be identical in appearance, smell and taste to the active agents. They were identical in their composition except for the active agents. The duration of the treatment in both arms is three years, in absence of concurrent illness that prevents further administration of treatment, an unacceptable adverse event, an unacceptable toxicity or if a patient decides to withdraw from the study. Treatment stops if general or specific changes in the patient's condition render the patient ineligible for further treatment in the judgment of the investigator or the treating physician.

#### Drug interactions/precautions

There are no known contra-indications, interactions or precautions for the use of selenium. A few individuals are allergic to yeast for whom this form of supplement would clearly be unsuitable, but this is not a common problem [19]. Known adverse health effects of selenium concern the synthesis of thyroid hormones (diminished T3 level), hepatotoxicity, gastrointestinal disturbances, nail and hair loss and dermatitis. However these symptoms have been associated with selenium intakes of more than 700 μg a day [31].

#### Safety and toxicity

Data on toxicity of selenium yeast are available from two animal studies while a number of human intervention studies show that the chronic administration of Se-yeast up to 800 μg/d provides no evidence of toxicity [19].

In 2008 there was an outbreak of selenium toxicity (selenosis). The source was identified as a liquid dietary supplement that contained 200 times the labelled concentration of selenium in the form of sodium selenite. Of 201 cases identified in ten states in the US, one person was hospitalized. The median estimated dose of selenium consumed was 41749 μg/day. Frequently reported symptoms included diarrhoea (78%), fatigue (75%), hair loss (72%), joint pain (70%), nail discoloration or brittleness (61%), and nausea (58%) [32]. It is suggested that hair, fingernails and toenails may all act as excretory organs when excess amounts of selenium are ingested [33]. The Food and Nutrition Board-Institute of Medicine [34] fixed the highest daily level of selenium that is likely to pose no risk of adverse health effects in almost all individuals at 400 μg selenium per day [31].

#### Compliance and drug accountability

The message of compliance is repeated during each follow-up visit. Patients are encouraged to return unused medication and empty packs. In order to check compliance, the research nurse counts and records the remaining unused tablets during each follow-up visit. Unused medication is collected and destroyed by PharmaNord.

#### Data collection

Patients receive a self-completion questionnaire after TUR, comprising questions about socio-demographics (age, sex, ethnicity, marital status, education) health-related lifestyle (lifetime smoking, history, passive smoking), medical and drug history, dietary intake (food-type frequency, alcohol, caffeine and total fluid intake, use of vitamins), social support and quality of life. The quality-of-life questions were developed by the European Organization for Research and Treatment for Cancer (EORTC). Assessments using the general cancer questionnaire QLQ-C30 and the QLQ-BLS24, with 24 supplemental questions, are made at baseline, after 6 and 36 months. A full version of the baseline and the follow-up questionnaires is available as a free download at http://www.bcpp.bham.ac.uk/seleblateng/patients.shtml. Patients' medical records are regularly examined by the research nurses for information on clinical treatment, histopathology, and outcome measures, which are reported on case report forms.

Blood samples are collected at baseline. A research nurse collects blood from all patients in a 5-mL EDTA tube for selenium measurement and one plain 10-mL tube for DNA-analysis. After the three-year intervention period a new blood sample for selenium measurement is collected.

#### Adverse event and serious adverse event monitoring strategy

For the purpose of this trial, any detrimental change in the patient's condition subsequent to the start of trial treatment and during the trial follow-up period, which is not unequivocally due to the underlying disease process of bladder cancer, is considered as either an adverse event (AE) or a serious adverse event (SAE). Adverse events and serious adverse events are being monitored during and between regular follow-up visits in accordance with the legal regulations.

#### Statistical analysis

Final statistical analysis is on an intention-to-treat basis. Kaplan-Meier estimates of a recurrence-free interval are used to compare treatment groups descriptively, whilst log-rank tests are used to test the hypothesis of no difference between treatments. Cox proportional hazards regression models are used to estimate the Hazard risks and 95% confidence intervals comparing patients randomized to active selenium versus placebo with and without adjustment for age, sex and stage at time of diagnosis. A per protocol analysis will be performed as a sensitivity analysis. We will also test for interactions with possible additional effect modifiers such as smoking, selenium at baseline as an indicator of selenium intake. For secondary outcome measures, treatment arms are compared in terms of progression-free interval using Kaplan-Meier and log-rank tests. The reasons for excluding patients from the analysis are clearly reported.

All analyzes are performed using STATA (StataCorp. 2009. *Stata Statistical Software: Release 11*. College Station, TX: StataCorp LP)

#### Sample size calculations

Sample size calculations are based on the recent meta-analysis (N = 2820) by Malström et al. [35] who looked at the recurrence after standard treatment (instillations of mitomycine or BCG). This is the group that corresponds best with our placebo-group (standard care). The absolute difference between the placebo- and the Selenium-group is based on the difference in percent of recurrence after five years (average of time after randomization in our study). A difference of 12,5% in absolute reduction can be expected, considering that the intake of 200 μg of SelenoPrecise yeast increases the plasma selenium by ± 100 ng/mL [29] and that, according to epidemiological studies, the incidence of bladder cancer decreases by 25% if the plasma selenium increases by 10 ng/mL [10].

To detect an absolute decrease of 12,5% in the recurrence-free rate by selenium versus placebo, 700 patients are needed, taking into account a drop out of 25% with a power of 86% (two-sided test).

#### Interim analyses

An independent data and safety monitoring board will periodically review the efficacy and safety data. Two formal interim analyses of efficacy will be performed as soon as the first 100 patients have reached one and two years after randomization. The interim analyses will enable the study to be stopped early if a clear result should emerge. This will take place if a statistically significant increase or decrease in recurrence rate of at least 10% has been found.

#### Baseline characteristics

At the time of writing we have recruited 177 patients. After one year of recruitment, 137 patients were assessed for eligibility of which 100 met the inclusion criteria. All patients were between 46 and 88 years and their mean age was 68 (± 9) years. Eighty-three percent of the patients were male and 87% of them Dutch-speaking. Sixty percent had a primary tumour and 60% had a solitary tumour. Seventy-nine percent of the tumours were papillary. The baseline plasma selenium was 84.8 ng/mL (± 18.5 ng/mL). Baseline characteristics were summarized in Table 1.

**Table 1:** Baseline characteristics of randomized participants in the SELEBLAT trial

Variable		Total 100	
Gender [N (%)]	Male [N (%)]	83	(83)
	Female	17	(17)
Age	≤ 50 yrs. [N (%)]	2	(2)
	51-60 [N (%)]	17	(17)
	61-70 [N (%)]	34	(34)
	71-80 [N (%)]	36	(36)
	> 80 [N (%)]	11	(11)
	Years [mean, (SD)]	68	(9)
Language [N (%)]	Dutch	87	(87)
	French	13	(13)
Primary	Primary	61	(60)
Tumour [N (%)]	Recurrent	35	(35)
	Unknown	5	(5)
Tumour	G1	31	(30)
Grade [N (%)]	G2	35	(34)
	G3	33	(32)
	Unknown	4	(4)
Tumour	pTa	60	(58)
Stage [N (%)]	pT1	31	(30)
	Cis	5	(5)
	Unknown	7	(7)
Tumour	Papillary	121	(79)
Type [N (%)]	Cis	29	(19)
	Unknown	3	(2)
Tumour	< 1 cm	59	(38)
Size [N (%)]	≥1 and ≤ 3	56	(37)
	> 3 cm	38	(25)
Tumour	Solitary	59	(60)
Number [N (%)]	2 or more	36	(36)
	Unknown	4	(4)
Selenium	ng/mL [mean, (SD)]	84.8	(18.5)

## Discussion

This is the first report of a trial in tertiary prevention of selenium for bladder cancer. Other chemopreventive trials have been performed [24,36]. High dose vitamins improved significantly the time to tumour recurrence (p = 0.0014) [37]. Retinoids have been tested with various results [38-40]. Neither vitamin B6 (Pyridoxine) [41,42] nor difluoromethylornithine reduced the rate of recurrence [43]. Lactobacillus casei powder significantly reduced the time to recurrence in the intervention group [44,45]. Several ongoing trials are investigating cyclooxygenase-2 inhibitors in the recurrence of bladder cancer. Preliminary analysis of the results of the study based at the MD Anderson Cancer Center did not demonstrate a difference in time to tumour recurrence between the two treatment arms [24]. We are aware of one other selenium and vitamin E factorial trial (SELENIB) that is currently recruiting in the UK [46]. This chemoprevention study, which is largerly similar to our study (as far as the selenium arm is concerned), aims to include 500 patients with non muscle-invasive bladder cancer. In this trial, patients receive a daily supplement of 200 μg high selenium yeast or placebo and 154 mg daily α-tocopherol or placebo for 5 years using a 2 × 2 factorial design. Two chemopreventive trials on prostate cancer with selenium yeast are currently ongoing [47].

Selenized yeast was selected for use in this trial because of its availability and well-characterized safety profile. In both the SELECT trial (prostate cancer prevention) and the NPC trial (basal cell or squamous cell skin cancer prevention), selenium supplementation was not significantly associated with any of the cardiovascular disease endpoints during 7.6 years of follow-up (all cardiovascular diseases, myocardial infarction, stroke, all cardiovascular disease mortality) [48]. In secondary analyses of data from the NPC study, 58 subjects in the selenium-supplemented group (n = 600) and 39 subjects in the placebo group (n = 602) developed type 2 diabetes (hazard ratio 1.55; 95% CI, 1.03-2.33) [49]. In the SELECT trial a statistically non-significant increased risk of type 2 diabetes was observed in the selenium group (RR, 1.07; 99% CI, 0.94-1.22) but not in the selenium vitamin E group [21]. A secondary analysis was carried out using data from a pre-existing randomized clinical trial designed to investigate the effects of selenium yeast on prostate cancer progression (Watchful Waiting Trial) [50]. There were no statistically significant differences in glucose levels during the course of the trial in men supplemented with selenium as compared with those on placebo. Laclaustra et al. reports that in U.S. adults, high serum selenium concentrations were associated with higher prevalence of diabetes and higher fasting plasma glucose and glycosylated hemoglobin levels. Mean serum selenium level in this trial was 137.1 ng/mL [51]. In the New England case-control study, independently of the selenium serum level, an increase bladder cancer risk was associated with a history of diabetes (adjusted odds ratio = 2.2, 95% CI, 1.3 to 3.8). The risk may be greater among patients taking oral hypoglycemics and those with diabetes of longer duration [52]. Total cholesterol, triglycerides, LDL cholesterol, and fasting serum glucose concentrations significantly increased with serum selenium concentration in the Taiwanese elderly. The mean serum selenium concentration was 89.76 ng/mL [53]. In the UK, the PRECISE Pilot trial randomized 501 elderly volunteers of relatively low selenium status [mean (SD) plasma selenium 88.8 (19.2 ng/mL)] to a six-month treatment with 100, 200 or 300 mg selenium/day as high-selenium yeast or placebo yeast [36]. Supplementation at 100 and 200 mg selenium/day lowered total serum cholesterol and non-HDL cholesterol [54]. The effects of a long-term selenium supplement on blood pressure are inconsistent. Laclaustra et al. reported that blood pressure increases if the plasma selenium level is higher than 160 ng/mL [55]. In a cross-sectional study with selenium in Belgium, the Flemish Study on Environment Genes and Health Outcomes (FLEMENGHO), 20 ng/mL higher plasma selenium level was associated with a clinically not relevant lower blood pressure with an effect sizes of 2.2 mmHg systolic (95% CI -0.57 to -5.05; p = 0.009) and 1.5 mmHg diastolic (95% CI -0.56 to -2.44; p = 0.017) in men, but not in women [56]. As in Belgium the daily intake of selenium is low, we think that patients taking selenium for three years can only benefit from this treatment, including of blood pressure reduction.

If it is possible to increase the time to recurrence with selenium, patients have to undergo fewer cystopscopies. This means a reduction not only in suffering for the patient, but also in costs for society.

We are aware that the selenium status at baseline and the genetic variation of tested individuals may represent additional reasons for positive or negative results [8] or risk of disease [54]. There are 25 selenoproteins and three selenium-containing enzymes (glutathione peroxidases, thioredoxin reductases and iodothyronine deiodinases), the first two being linked with antioxidant activity, and the latter involved with thyroid hormone metabolism. Rayman [57] suggests that only those persons at risk, those with single nucleotide polymorphisms in selenoproteins, GPx1, GPx4, SEPS1, Sep15, SEPP1 and TXNRD1, should be treated with selenium at doses which optimize the plasma selenium levels in order to activate specific selenoproteins. An intake of 40 μg per day is required to maintain the plasma glutathione peroxidase (GPx) activity at plateau [58]. We will be investigating those additional genetic analyses in a subsequent sub-project.

The SELEBLAT study is well-designed. Selenium-yeast is a safe and cheap medicine. A computerized program performs our randomization. Neither the investigators, nor the research nurses have access to the randomization numbers. All study personnel is blinded for the medication. Both treatment groups are regularly compared on similarity of prognostic characteristics such as age, gender, smoking, grade of tumour, baseline selenium level and co-morbidity. We will report reasons and numbers of those patients lost to follow up. Both an intention to treat and a per protocol analysis will be performed. For hypothesis building subgroup analyses and analyses of interactions will be performed, as we have demographic and clinical data and questionnaires results on diet and Quality of Life. Standard treatment will be compared between the intervention and the placebo group. Additional intake of selenium by food supplements will be analyzed and compliance will be reported. Our primary outcome measures are both clinical and histologically confirmed recurrences as frequently patients with clinical recurrence are only cauterized without taking a biopsy. Cauterization only reduces the risk of perforation of the bladder. All subsequent patients undergoing TUR operation are included meeting the inclusion criteria. We decided to include both primary and second primary tumours, because the incidence of bladder cancer is low, while the prevalence and the rate of tumour recurrence is high [24]. Recurrence within the first three months after TUR is considered as residual untreated disease. Although bladder cancer and prostate cancer occur together in 10% of the cases [59,60], we decided to exclude patients with prostate cancer too. We deliberately did not exclude any patient for presence of co-morbidity or age aiming for a study population, which represents as far as possible the normal population diagnosed with bladder cancer. We intend to reach the calculated sample size by pooling of our results with the results from the Selenib study (UK), which is similar in design. Progress of the study can be followed on the website: http://www.genepid.bham.ac.uk/Seleblat_Recruitment.shtml. The first results of our study can be expected for 2014.

## Conflict of interests

The authors declare that they have no competing interests.

## Authors' contributions

MG: study design, conception and design of the article, analysis and interpretation of data, drafting the article. FB: study design, conception and design of the article, analysis and interpretation of data, drafting the article. SJ: study design, revising the article critically for important intellectual content. KA: local investigator, recruitment of patients, revising the article critically for important intellectual content. FA: local investigator, recruitment of patients, revising the article critically for important intellectual content. IB: local investigator, recruitment of patients, revising the article critically for important intellectual content. JB: local investigator, recruitment of patients, revising the article critically for important intellectual content. AB: local investigator, recruitment of patients, revising the article critically for important intellectual content. JD: local investigator, recruitment of patients, revising the article critically for important intellectual content. KD: local investigator, recruitment of patients, revising the article critically for important intellectual content. LG: local investigator, recruitment of patients, revising the article critically for important intellectual content. EK: study design, revising the article critically for important intellectual content. BT: local investigator, recruitment of patients, revising the article critically for important intellectual content. SV: recruitment of patients, revising the article critically for important intellectual content. BV: recruitment of patients, revising the article critically for important intellectual content. FV: local investigator, recruitment of patients, revising the article critically for important intellectual content. KV: local investigator, recruitment of patients, revising the article critically for important intellectual content. HV: study design, revising the article critically for important intellectual content. MZ: study design, conception and design of the article, analysis and interpretation of data, drafting the article. All authors read and approved the final manuscript.

## Pre-publication history

The pre-publication history for this paper can be accessed here:

http://www.biomedcentral.com/1471-2490/12/8/prepub
